# Molecular phylogenetic reconstruction and localization of the (TTAGG)n telomeric repeats in the chromosomes of *Acromyrmex striatus* (Roger, 1863) suggests a lower ancestral karyotype for leafcutter ants (Hymenoptera)

**DOI:** 10.3897/CompCytogen.v12i1.21799

**Published:** 2018-01-09

**Authors:** Tássia Tatiane Pontes Pereira, Ana Caroline Coelho Corrêa dos Reis, Danon Clemes Cardoso, Maykon Passos Cristiano

**Affiliations:** 1 Departamento de Biodiversidade, Evolução e Meio Ambiente, Universidade Federal de Ouro Preto, Ouro Preto, MG, Brazil; 2 Programa de Pós-graduação em Genética, Universidade Federal do Paraná, Curitiba, PR, Brazil

**Keywords:** fluorescence in situ hybridization (FISH), telomere, phylogenetic reconstruction, chromosome evolution, Formicidae

## Abstract

Chromosome counts and karyotype characterization have proved to be important features of a genome. Chromosome changes during the diversification of ants might play an important role, given the diversity and success of Formicidae. Comparative karyotype analyses on ants have enriched and helped ant systematics. Among leafcutter ants, two major chromosome counts have been described, one frequent in *Atta* Fabricius, 1804 (2n = 22 in all *Atta* spp. whose karyotype is known) and the other frequent in *Acromyrmex* Mayr, 1865 (2n = 38 in the majority of species whose karyotype is known). The main exception is *Acromyrmex striatus* (Roger, 1863), which harbors a diploid chromosome set of 22. Here we describe the use of fluorescence in situ hybridization (FISH) with telomeric probes with (TTAGG)_6_ repeats to describe the telomere composition of *A. striatus* and to recover potential interstitial non-telomeric signals that may reflect fusion events during the evolution of leafcutter lineage from 38 to 22 chromosomes. Further, we reconstruct the ancestral chromosome numbers of the leafcutter clade based on a recently proposed molecular phylogenetic hypothesis and phylogenomic tree. Distinct signals have been observed in both extremities on the telomere chromosomes of *A. striatus*. Non-telomeric signals have not been retrieved in our analysis. It could be supposed that the low-numbered karyotype indeed represents the ancestral chromosome number of leafcutters. The phylogenetic reconstruction also recovered a low chromosome number from the diverse approaches implemented, suggesting that n = 11 is the most likely ancestral karyotype of the leafcutter ants and is a plesiomorphic feature shared between *A. striatus* and *Atta* spp.

## Introduction

The nuclear genome of any eukaryote is confined within the chromosomes, which vary in number, size, and shape. In turn, macromolecular structures, such as centromeres and telomeres, can be cytologically distinguished on each chromosome (Lysak and Schubert 2013). These latter terminal structures on the chromosomes are composed of tandem repeats that usually prevent the loss of DNA during replication, thereby promoting their stability. The telomere sequences are conserved across the species of a particular group. Among insects, the most common telomeric sequence reported is (TTAGG)_n_, but that is not a general consensus (Frydrychová et al. 2004). Among ants, it has been confirmed by fluorescence in situ hybridization (FISH) and by the Southern blotting technique as being the most frequent repeat in Dolichoderinae, Formicinae, and Myrmicinae subfamilies (Lorite et al. 2002).

Several studies have correlated the presence of non-telomeric signals or interstitial telomeric signals as evidence that the chromosomes have undergone structural and/or numerical rearrangements. For instance, after a Robertsonian chromosome fusion, the telomeric sequences might remain in interstitial sites of this new fused chromosome and can be detected today. Interstitial telomeric signals have been detected on the chromosomes of different animal groups, such as mammals (Ventura et al. 2006), fishes (Bitencourt et al. 2014), and insects (Šíchová et al. 2016). Among ants, the localization of (TTAGG)_n_ telomeric repeats on the chromosomes was carried out only with the well-known bulldog ants from the genus *Myrmecia* Fabricius, 1804 (Meyne et al. 1995), and on the chromosome set (2n = 18) of the ant species *Tapinoma nigerrimum* (Nylander, 1856) (Lorite et al. 2002). In both studies, positive hybridization signals were observed on the telomeres of the chromosomes. Lorite et al. (2002) suggested that the telomeric repeat (TTAGG)_n_ is conserved among the ant lineages. It has been recently suggested that this telomere repeat was likely lost in the ancestor of Apocrita and is putatively regained in Formicidae and Apidae, since only they comprise species in which this motif was detected (Menezes et al. 2017).

Ants comprise a natural and diverse group consisting of more than 16,000 species (Bolton 2017). In the Nearctic and Neotropics, but mainly in the latter, the leafcutter ants of the genera *Atta* Fabricius, 1804 and *Acromyrmex* Mayr, 1865 stand out, which, together, with all other 15 genera of fungus-farming ants, cultivate crops of symbiotic Basidiomycete fungi inside their nests. Cytogenetic data for leafcutter ants are available, so far, for 21 species comprising *Atta* (five spp.) and *Acromyrmex* (16 spp. including subspecies and/or varieties). All *Atta* species (five cytogenetically studied out of 17 valid species) display karyotype uniformity with a diploid set of 22 chromosomes and karyotypic formula of 2n = 18M + 4A, except *Atta robusta* Borgmeier, 1939, whose karyotypic formula is 2n = 18M + 2SM + 2ST (Barros et al. 2015). Yet, *Acromyrmex* (14 species and subspecies cytogenetically studied out of 62) display a slightly higher uniformity in the chromosome counts, with a diploid set of 38 chromosomes, but a very large variability in its chromosome morphology, bearing distinct karyotypic structures (Barros et al. 2016), suggesting a structural dynamic nature of the genome of *Acromyrmex* lineages. Two chromosome counts diverge from the 2n = 38 common chromosome number, the first from *Ac. ameliae* de Souza, Soares & Della Lucia, 2007 (2n = 36) (Barros 2010), and the second much more pronounced from *Ac. striatus* (Roger, 1863) (2n = 22) (Cristiano et al. 2010), the same chromosome number as *Atta* spp. (Cristiano et al. 2013). It has been proved by molecular phylogenetic analysis that *A. striatus* is a sister group of the remaining leafcutter ants (Cristiano et al. 2013), which was later confirmed by phylogenomic analysis (Branstetter et al. 2017). Although *A. striatus* and *Atta* spp. shared some morphological characteristics and the same number of chromosomes, they differ in some karyotype features (Cristiano et al. 2013, Barros et al. 2015).

Given the phylogenetic position of *A. striatus* and chromosome evolution based on a phylogenetic approach, it has been supposed that n = 11 is the ancestral chromosome number of leafcutter ants (Cristiano et al. 2013). Here, we investigate the position of telomeric signals by means of FISH with telomeric probes (TTAGG)_n_ in order to detect telomeric and/or non-telomeric hybridization signals on the chromosomes of *A. striatus*, in order to dismiss the alternative hypothesis that *A. striatus* karyotype arose by chromosome fusion due to the uniform chromosome number in the genus. We tested the hypothesis that no interstitial markers would be observed given that the haploid chromosome number of 11 chromosomes of *A. striatus* is the ancestral karyotype of the leafcutter ants. Further, we tested once more the ancestral chromosome number of all leafcutter ants by using the renewed phylogenetic approach to ancestral reconstruction CHROMEVOL 2.0 (Glick and Mayrose 2014) to estimate the potential ancestral chromosome number of leafcutter ants based on a phylogenetic tree with a more comprehensive phylogenetic and cytogenetic matrix.

## Material and methods

### Sampling, chromosome preparations and fluorescence *in situ* hybridization

Colonies were sampled from restinga environments of Morro dos Conventos, Araranguá–Santa Catarina, Brazil (S28°56'08.2', W49°21'28") (permit SISBIO-ICMBio 45464-1), transferred to the Laboratório de Genética Evolutiva e de Populações of the Universidade Federal de Ouro Preto–MG, and maintained as described by Cardoso et al. (2011) until obtaining a brood. Metaphase chromosomes were obtained from 20 individuals according to the protocol established by Imai et al. (1988), with modifications described in Cardoso et al. (2012), using the ganglia of prepupae. Metaphase spreads were conventionally stained with Giemsa and with Fluoroshield with DAPI (Sigma-Aldrich, St. Louis, Missouri, USA) for chromosome counting and karyomorphometry as described by Cristiano et al. (2017).

Telomeric FISH was carried out according to the procedure described by Cioffi et al. (2011) on seven slides where the best metaphase spreads and an average number of ten metaphases were analyzed. The telomeric probe (TTAGG)_6_ was directly labeled with Cy3 on the 5’ end. Briefly, metaphase spreads were denatured for 5 min in 70%/2xSSC formamide at 75 °C. Probes were hybridized with chromosomes in 20 µl of hybridization mix containing the following: 200 ng of labeled probe, 50% formamide, 2xSSC, and 10% dextran and sulfate 20xSSC. This hybridization mix was heated for 10 min at 85 °C; slides were kept in a moist chamber at 37 °C overnight. Then, slides were washed in 4xSSC/Tween and dehydrated in a series of alcohol solutions. Slides were mounted in antifade solution with DAPI (DAPI Fluoroshield, Sigma-Aldrich). The DAPI-stained slides were then analyzed under an Olympus BX53 epifluorescence microscope coupled to an MX10 digital camera using cellSens imaging software.

### Phylogenetic analysis and ancestral state inference

The ant DNA sequences were obtained from GenBank, representing the matrix data from Cristiano et al. (2013) and Cardoso et al. (2014a, b), with some sequences originally obtained from Schultz and Brady (2008). Four genomic fragments were included: *wingless*, *long-wavelength rhodopsin*, *elongation factor 1-alpha paralog F1* and *paralog F2*. The sequences were aligned visually in MEGA v7 (Kumar et al. 2016). Ambiguously aligned sites were excluded (i.e., the *long-wavelength* intronic region) and the alignment was confirmed by translation to amino acids, whereas the missing data were coded as “?”.

The phylogenetic inference was carried out by using Bayesian methods with Markov Chain Monte Carlo (MCMC) methods. In order to select the substitution model of DNA evolution that fits best to each potential partition under Akaike’s Information Criterion (AIC) and Bayesian Information Criterion (BIC), we used PARTITIONFINDER2 (Lanfear et al. 2014, 2017). Taking into account the estimated parameters, we carried out a Bayesian analysis in MRBAYES v3.2.6 (Ronquist and Huelsenbeck 2003), which consisted of two independent runs of twenty million generations each, sampled every 1000 generations, and the convergence between runs was determined using TRACER v1.4 (Rambaut and Drummond 2007). A burn-in period, in which the initial 25% of the trees were discarded, was adopted to produce a consensus topology that was visualized using the FIGTREE V1.4 program (Rambaut 2009). The consensus topologies inferred were implemented in the inference of ancestral chromosome numbers at internal nodes.

In order to estimate the ancestral haploid chromosome number of the leafcutter ants and all remaining internal nodes, we carried out three independent analyses using CHROMEVOL 2.0 (Glick and Mayrose 2014). We used this integrative cytogenetic and molecular phylogeny approach by using two probabilistic methods, maximum likelihood (ML) and Bayesian inference (BI), to infer the chromosome evolution model and haploid ancestral states (haploid chromosome number at internal nodes), relying on a phylogenetic hypothesis estimated using the matrix and models described above. The new data matrix included about 50% (20 spp.) more species whose chromosome counts are available.

CHROMEVOL 2.0, under ML and BI inference, evaluates ten chromosome evolution models and different transitions between chromosome numbers. Basically, models evaluate dysploidy (decrease or increase by a single chromosome number in the haploid set of chromosomes, constant or linear, the latter being dependent on the current chromosome number), polyploidy (duplication of whole chromosome complement), and demi-polyploidy (the process that allows karyotypes with multiples of a haploid karyotype). The latter mechanism allows the transition from a haploid karyotype (n) to 1.5n, which could be possible in ants if related species hybridize due to the haplodiploid genetic system. Yet, polyploidy could be more unlikely. Although it is widespread and common in plants, polyploidization occurs very rarely in animals due to various incompatibility problems, so models with this parameter were not evaluated. All parameters were adjusted for the data, as described by Mayrose et al. (2010) and Glick and Mayrose (2014), and as performed by Cristiano et al. (2013) and Cardoso et al. (2014). The model that, to date, fits best, and the null hypothesis of no duplication, were analyzed with 10,000 simulations under the AIC. Further, to check for possible inconsistencies in the ancestral reconstruction due to the topologies recovered in our phylogenetic analysis, an additional run was performed by using the phylogenomic tree of Branstetter et al. (2017). Then, the same chromosome number matrix was implemented by adding the information from the new taxa when available, to meet the taxa comprising the phylogenomic tree.

## Results

**Figure 1.**
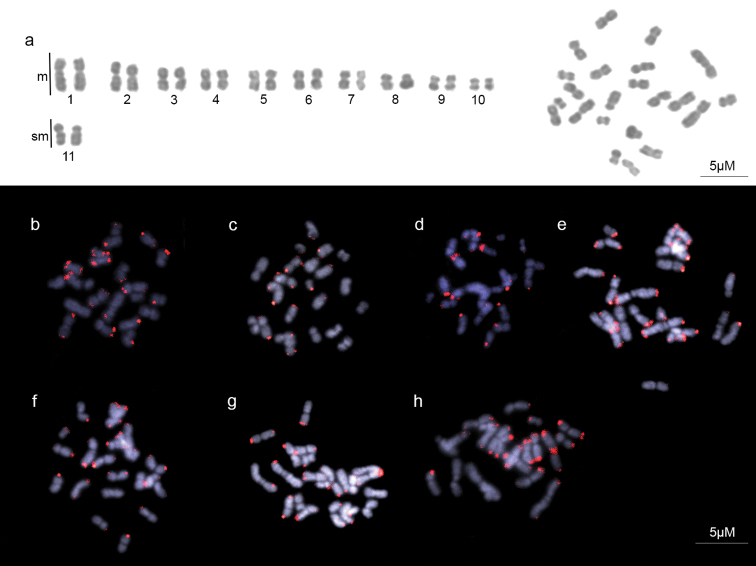
Metaphase and karyotype of *A. striatus* and metaphase spreads after FISH with the telomeric probe (TTAGG)_6_. **a** Metaphase and karyotype stained with Giemsa **b–h** Best metaphase spreads stained with DAPI (uniform blue) and the telomeric probes with Cy3-dUTP in red.

The chromosome counts for all individuals of *A. striatus* analyzed here were 2n = 22 (Figure 1a). The karyotype of this species consists of 10 metacentric (M) pairs and one submetacentric (SM) pair. Thus, the karyotypic formula of the diploid set was 2K = 20M + 2SM and the fundamental number was FN = 44. Morphometric data for chromosomes were confirmed and are presented in Table 1. We were able to detect the telomere sequences (TTAGG)_6_ by FISH (Figure 1b–h). Positive signals were recovered at both ends of the chromosomes of *A. striatus*; however, the size and intensity of the signals varied among the terminal telomeric portions of the chromosomes, and between metaphase spreads (Figure 1b–h). No signals for interstitial telomeric sites were detected.

**Table 1.** Karyomorphometric analyses of the chromosomes of *Acromyrmex striatus* from ten well-spread metaphases.

Chromosome	TL	L	S	RL	*r*	Classification
1(a)	4.34±0.62	2.58±0.41	1.67±0.21	7.01±0.34	1.55±0.16	Metacentric
2(a)	3.98±0.65	2.33±0.45	1.59±0.23	6.42±0.45	1.46±0.17	Metacentric
3(b)	3.66±0.64	2.1±0.46	1.52±0.23	5.9±0.53	1.37±0.17	Metacentric
4(b)	3.43±0.52	1.89±0.31	1.47±0.19	5.54±0.37	1.28±0.11	Metacentric
5(c)	3.17±0.5	1.73±0.31	1.4±0.22	5.11±0.27	1.24±0.18	Metacentric
6(c)	2.98±0.39	1.6±0.23	1.36±0.17	4.82±0.1	1.18±0.1	Metacentric
7(d)	2.94±0.38	1.56±0.22	1.31±0.18	4.76±0.1	1.19±0.07	Metacentric
8(d)	2.87±0.37	1.57±0.23	1.28±0.18	4.63±0.12	1.25±0.19	Metacentric
9(e)	2.82±0.33	1.56±0.2	1.18±0.19	4.56±0.12	1.35±0.21	Metacentric
10(e)	2.76±0.32	1.5±0.21	1.24±0.15	4.47±0.13	1.22±0.14	Metacentric
11(f)	2.72±0.31	1.54±0.19	1.17±0.17	4.4±0.1	1.33±0.15	Metacentric
12(f)	2.66±0.29	1.44±0.21	1.23±0.12	4.32±0.1	1.17±0.09	Metacentric
13(g)	2.62±0.3	1.49±0.27	1.14±0.11	4.24±0.11	1.31±0.15	Metacentric
14(g)	2.56±0.31	1.41±0.18	1.13±0.13	4.14±0.09	1.25±0.13	Metacentric
15(h)	2.45±0.35	1.35±0.22	1.06±0.14	3.95±0.18	1.27±0.12	Metacentric
16(h)	2.33±0.32	1.25±0.21	1.02±0.16	3.76±0.16	1.23±0.09	Metacentric
17(i)	2.07±0.17	1.09±0.14	0.92±0.13	3.37±0.18	1.2±0.17	Metacentric
18(i)	1.95±0.13	1.06±0.1	0.88±0.12	3.17±0.19	1.21±0.12	Metacentric
19(j)	1.75±0.16	0.9±0.11	0.75±0.07	2.84±0.15	1.2±0.09	Metacentric
20(j)	1.59±0.18	0.83±0.11	0.69±0.09	2.57±0.17	1.21±0.07	Metacentric
21(k)	3.2±0.55	2.16±0.35	0.96±0.18	5.23±1.1	2.27±0.27	Submetacentric
22(k)	2.94±0.43	2.02±0.3	0.93±0.16	4.8±0.74	2.18±0.25	Submetacentric
∑	61.79					

***TL***: total length; ***L***: long arm length; ***S***: short arm length; ***RL***: relative length; ***r***: arm ratio (= *L*/*S*).

**Figure 2.**
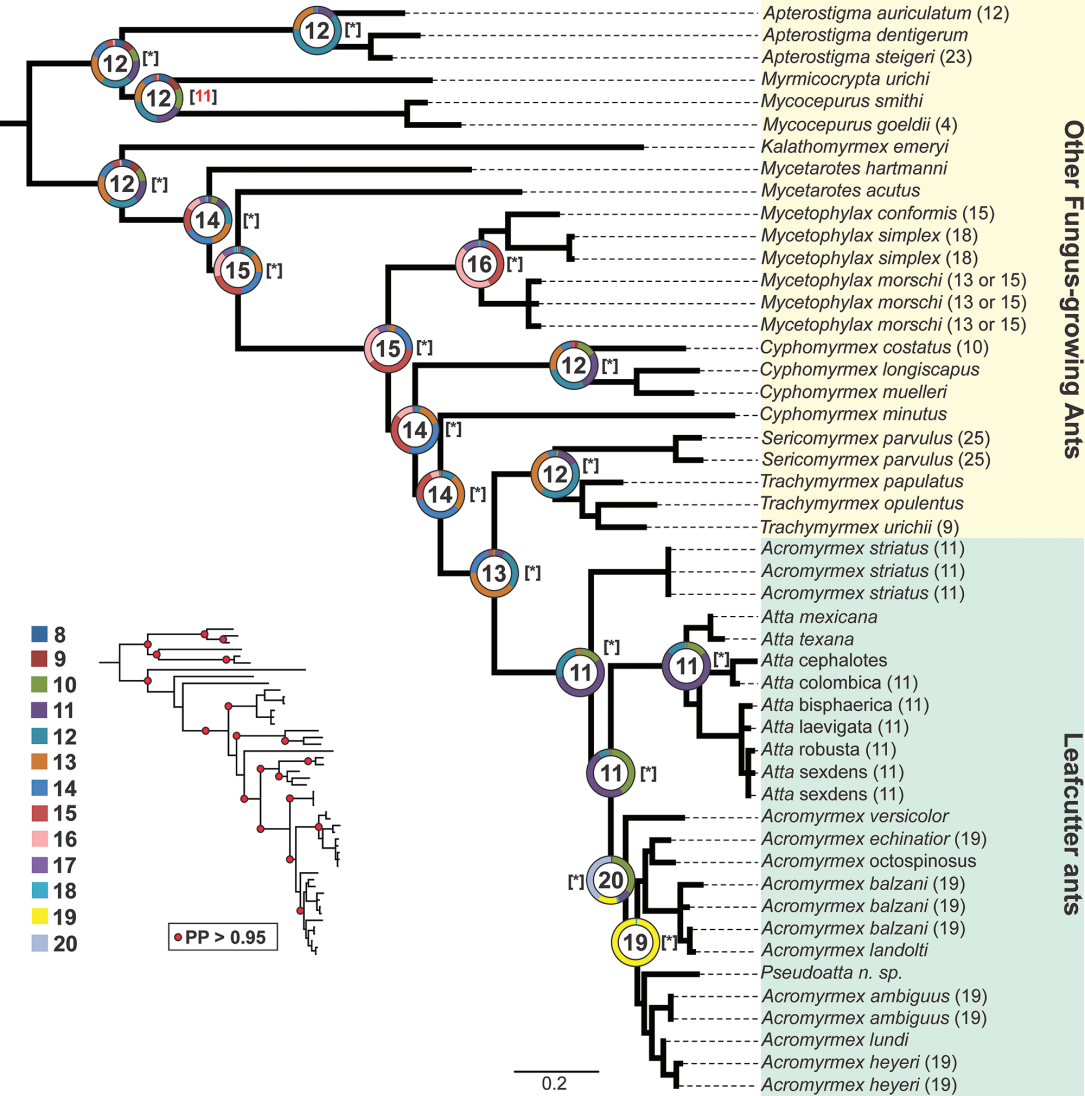
Ancestral haploid chromosome state reconstruction inferred under Bayesian Inference and Maximum Likelihood methods. The ancestral chromosome number with the highest probability is given inside the circle and pie charts at the main nodes. The colors on the pie charts represent the proportional probability of each given chromosome number according to the legend. The known karyotypes of species are given at the tip. The haploid ancestral chromosome numbers with the best likelihood are given in brackets. * represent the same estimated haploid number in BI.

An alignment of 1796 base pairs was obtained for the four concatenated nuclear genes comprising 49 sequences of fungus-growing ants, whose species from the genera *Apterostigma* Mayr, 1865, *Mycocepurus* Forel, 1893 and *Mycetarotes* Emery, 1913 were placed as outgroups. Nine different substitution models were estimated by PARTITIONFINDER2 for each gene codon position (see Table 2 for details on the partition scheme) and were employed in the Bayesian analysis. As expected, all species of the *Acromyrmex* and *Atta* genera formed a well-supported clade that fell as a sister group of *A. striatus*. The results obtained in the analysis of chromosome evolution suggested that the best-supported model of the process underpinning chromosome change was the hypothesis with constant gain, loss, and duplication (likelihood = –58.53, AIC = 123.1). These results revealed the possible occurrence of duplication events or an increase of the chromosome number by the whole genome duplication in the chromosome evolution of these species. However, the main events inferred were loss (fusion) and gain (fission), which showed PP > 0.5. In the Bayesian analysis, the haploid chromosome number at the most recent common ancestor (MRCA) of leafcutter ants with the highest posterior probability (PP) was n = 11. Likewise, in the ML analysis the most likely haploid number was n = 11 (Figure 2). The same results were observed in the analysis based on the phylogenomic tree, under both estimation approaches (Suppl. material 1: Figure S1).

**Table 2.** Models of molecular evolution by genes and codons implemented in the Bayesian analyses to infer the molecular phylogeny of fungus-growing ants. This tree was the topology inputted in CHROMEVOL 2.0 to estimate the ancestral chromosome numbers.

Gene (number of base pairs)	Position	Model
*wingless* (411bp)	1st – first position	K81+G
	2nd – second position	TIM+I+G
	3rd – third position	GTR+G
*elongation factor-1 alpha* F1 (402 bp)	1st – first position	TIM+I+G
	2nd – second position	GTR+G
	3rd – third position	GTR+G
*elongation factor-1 alpha* F2 (519 bp)	1st – first position	TIM+I+G
	2nd – second position	GTR+G
	3rd – third position	HKY+G
*long-wavelength rhodopsin* (464 bp)	1st – first position	SYM+I+G
	2nd – second position	GTR+I+G
	3rd – third position	TVM+G

## Discussion

All individuals of *A. striatus* from the population evaluated here had chromosome counts of 2n = 22. The karyotype of this species consists of 20 metacentric (M) and two submetacentric (SM) chromosomes, as reported by Cristiano et al. (2013). No difference in karyotype was expected since the samples analyzed here belong to closely related populations analyzed previously. However, differences in karyotype number within the same species are fairly likely among ants (Meyne et al. 1995, Imai et al. (1988), Cardoso et al. 2014b). No variation in the karyotypic formula was observed and the diploid set was 2K = 20M + 2SM, therefore, the fundamental number that corresponded to the number of chromosome arms in the diploid karyotype was FN = 44.

The (TTAGG)_6_ probe hybridized to both ends of chromosomes of *A. striatus*. This reveals the composition of the telomeric portions on chromosomes of the leafcutter ant *A. striatus*. The presence of the repeat (TTAGG)_6_ at the telomeres has already been reported in Apidae and Formicidae (Frydrychová et al. 2004) including ants *Tapinoma nigerrimum* and *Myrmecia* spp. (Lorite et al. 2002, Meyne et al. 1995). However, the TTAGG telomere motif was not detected in many other Hymenoptera, suggesting that it may have been lost and only regained in Apidae and Formicidae (Menezes et al. 2017). Nevertheless, the authors do not discard multiple losses along hymenopteran evolutionary history. Within Formicidae, the TTAGG motif was confirmed by Southern blot hybridization against digested genomic DNA in ant species from the Dolichoderinae, Formicinae, and Myrmicinae subfamilies (Lorite et al. 2002), suggesting that the telomeres of ants may be mainly comprised of (TTAGG)_n_. However, positive signals using the common vertebrate repeat (TTAGGG)_n_ were also detected by Meyne et al. (1995) on the chromosomes of *Myrmecia* spp. under low stringency (reduced percentage of formamide, less than 50%). Thus, the authors did not reject that both repeats might occur on ant chromosomes (Meyne et al. 1995). In fact, Lorite et al. (2002) emphasize that the main telomere sequence in ants is (TTAGG)_n_ instead of (TTAGGG)_n_, and that the latter should be present in very low copy number considering the Southern blot hybridization results. Here, we reported for the first time telomeric sequences by FISH in a neotropical leafcutter ant, adding to cytogenetic knowledge on this important insect group, and helping us to identify trends in ant chromosome evolution.

The size and intensity of (TTAGG)_6_ probe signals varied along the termini of the metaphase spreads of *A. striatus* (see Figure 1b-h). This was also observed in both studies that have performed telomeric FISH on ant chromosomes. Meyne et al. (1995) detected differential signals among termini of *Myrmecia* spp. by using both repeats mentioned above; this pattern is quite evident in *M. croslandi* Taylor, 1991 and *M. haskinsorum* Taylor, 2015. This variation in the hybridization signals was also observed on the chromosomes of *T. nigerrimum* (Lorite et al. 2002). We assume that this variation may be the result of two non-excluding processes. The first can be attributed to the difference in the number of telomeric repeats comprising the chromosomes, the second to the impairing of the probe hybridization as a result of protocol limitations in the chromosome preparation. In fact, when different metaphase spreads are evaluated, positive signals are detected over the negative signals on the chromosome of the next spread. Further, if positive signals have never been observed on dozens of metaphases, as recently reported for parasitoid Hymenoptera (Gokhman et al. 2014), this may represent an absence of signal of the evaluated telomeric repeat. Moreover, a negative signal on all chromosomes, as well as on all metaphases analyzed, was also reported in the neotropical wasp *Metapolybia decorata* (Gribodo, 1896) of the family Vespidae (Menezes et al. 2013), but this was not the case here.

In several species, the presence of interstitial telomeric signals on chromosomes has been observed (Meyne et al. 1990). These have been correlated with chromosome rearrangements and then used as markers of karyotype evolution and, consequently, may be used to evaluate the phylogenetic relationship of species, and even populations (Meyne et al. 1995, Quing et al. 2013). No interstitial telomere sequences were detected on the chromosomes of *A. striatus*. Thus, it could be supposed that the chromosome number observed in this species may represent the ancestral chromosome number of leafcutter ants. We do not ignore the fact that, due to the limitation of the FISH technique’s resolution, small interstitial telomeric signals originated by chromosomal fusions were not detected. However, the results of the cyto-phylogenetic approach to estimate the ancestral chromosome number have recovered the haploid number of 11 chromosomes in ML and BI (see Figures 2 and Suppl. material 1: Figure S1), in accordance with the previous estimate by Cristiano et al. (2013). This new estimate is based on 20 cytogenetic data values of fungus-growing ants instead of 12. Here, more cytogenetic and molecular data were added. However, a more detailed chromosome evolution hypothesis for fungus-growing ants, instead of just estimating the ancestral karyotype, will be possible when more simultaneous cytogenetic and molecular data are available. Chromosome evolution hypotheses based on cytogenetics coupled with molecular phylogenetic data were already drawn on the fungus-growing ant lineage from the genus *Mycetophylax* Emery, 1913, where fusion events were evidenced as having taken place during its karyotype evolution (Cardoso et al. 2014).

Likewise, the ancestral haploid chromosome number of 11 was recovered based on the phylogenomic tree of fungus-growing ants from Branstetter et al. (2017). On this tree, several non-fungus-growing outgroups were included, as well as other fungus-growing ants, but comprising the species included in the matrix of four nuclear genes used in the molecular phylogeny presented here. Together, telomeric FISH analysis of the karyotype of *A. striatus* and chromosomal reconstruction under two phylogenetic hypotheses based on independent data reinforce a low chromosome number as the putative ancestral karyotype for leafcutter ants. In fact, known karyotypes of *Trachymyrmex* Forel, 1893, the sister group to leafcutter ants, show as few as nine and 10 chromosomes in the haploid chromosome set (Murakami et al. 1998, Barros et al 2013).

Overall, in light of the results reported here, it is important to note that the evolution of the remaining *Acromyrmex* species, in contrast to the *Atta* spp. and *A. striatus* lineages, was mainly driven by the increase of chromosome number by centric fission. This could be followed by structural events, which, based on chromosome banding techniques, was suggested by Barros et al. (2016). Thus, the diploid number of 38 chromosomes, and likely 36 chromosomes in *A. ameliae*, represents a derived feature of the lineage leading to all other *Acromyrmex* species. Yet, in the lineage leading to *A. striatus* and *Atta* spp., the maintained number of 22 chromosomes may represent a plesiomorphic feature of their karyotypes.

## Supplementary materials

Supplementary material 1 Figure S1. Phylogenomic tree used to estimate the ancestral chromosome number. Data type: species data Explanation note: Numbers at nodes represent the first and second most likely haploid chromosome number followed by posterior support values under Bayesian optimization and the ancestral haploid chromosome number with best likelihood under maximum likelihood optimization, as follows: [first haploid state (P.P.%)// second haploid state (P.P.%)// ML haploid state]. This is an open access article distributed under the terms of the Creative Commons Attribution License (CC BY 4.0), which permits unrestricted use, distribution, and reproduction in any medium, provided the original author and source are credited.Tássia Tatiane Pontes Pereira, Ana Caroline Coelho Corrêa dos Reis, Danon Clemes Cardoso, Maykon Passos Cristiano
