# Supplementary material for: Molecular phylogenetic reconstruction and localization of the (TTAGG)n telomeric repeats in the chromosomes of Acromyrmex striatus (Roger, 1863) suggests a lower ancestral karyotype for leafcutter ants (Hymenoptera)
Source: Comp Cytogenet. 2018 Jan 9;12(1):13–21. doi: 10.3897/CompCytogen.v12i1.21799 (PMC5770561; doi:10.3897/CompCytogen.v12i1.21799)
Supplement: Supplementary material 1 — Figure S1. Phylogenomic tree used to estimate the ancestral chromosome number. [file comparative_cytogenetics-12-013-s001.docx]

**
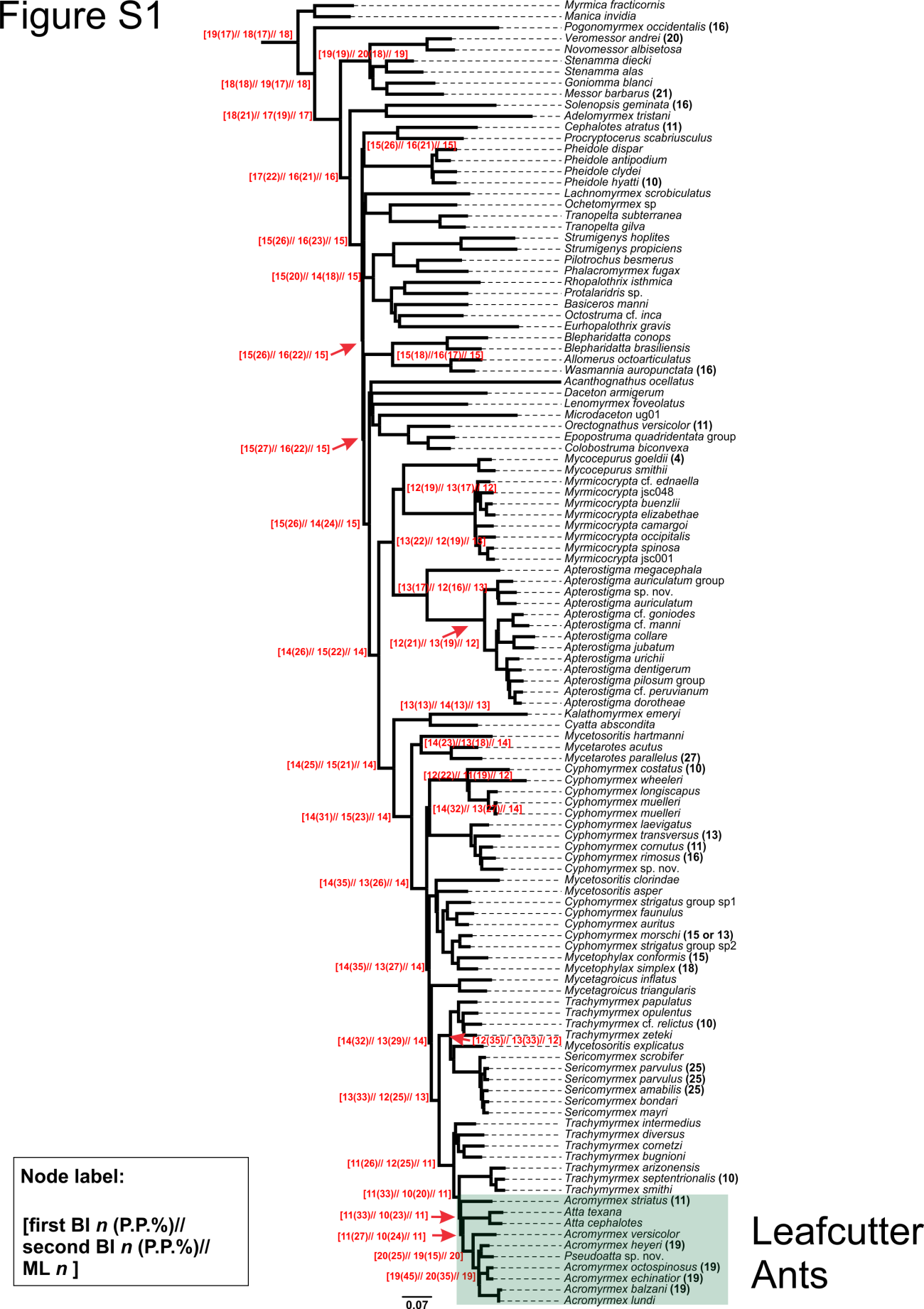
**

**Figure S1** – Phylogenomic tree used to estimate the ancestral chromosome number. Numbers at nodes represent the first and second most likely haploid chromosome number followed by posterior support values under Bayesian optimization and the ancestral haploid chromosome number with best likelihood under Maximum Likelihood optimization, as follow: [first haploid state (P.P.%)// second haploid state (P.P.%)// ML haploid state].
